# Análise da Fibrose Miocárdica Intersticial por Histologia e Ressonância Magnética Cardíaca na Valvopatia Aórtica de Grau Importante

**DOI:** 10.36660/abc.20250203

**Published:** 2026-04-16

**Authors:** Juliana Hiromi Silva Matsumoto Bello, Lea M.M.F. Demarchi, Rafael Almeida Fonseca, Gabriele N. Lima, Lucas José Tachotti Pires, Vitor E. E. Rosa, Roney Orismar Sampaio, Paulo S. Gutierrez, Vera D. Aiello, Flávio Tarasoutchi, Carlos E. Rochitte

**Affiliations:** 1 Hospital das Clínicas da Faculdade de Medicina da Universidade de São Paulo Instituto do Coração São Paulo SP Brazil Instituto do Coração do Hospital das Clínicas da Faculdade de Medicina da Universidade de São Paulo, São Paulo, SP – Brazil; 2 Hospital das Clínicas da Faculdade de Medicina da Universidade de São Paulo São Paulo SP Brazil Hospital das Clínicas da Faculdade de Medicina da Universidade de São Paulo, São Paulo, SP – Brazil

**Keywords:** Valvopatia Aórtica, Miocárdio, Fibrose, Imageamento por Ressonância Magnética, Histologia

## Abstract

**Fundamento:**

A valvopatia aórtica pode levar ao acúmulo de fibrose miocárdica (FM). A ressonância magnética cardíaca (RMC) com realce tardio (RT) avalia a fibrose regional, mas não detecta a fibrose intersticial, que pode ser estimada pelo volume extracelular (VEC) utilizando T1 nativo (pré-contraste) e pós-contraste.

**Objetivos:**

Comparar a FM medida pela fração de volume de colágeno (FVC) em biópsia miocárdica com RT e VEC em pacientes com valvopatia aórtica de grau importante.

**Métodos:**

Um total de 108 pacientes submetidos à troca valvar aórtica com biópsia miocárdica e RMC pré-operatória foi incluído. RT e VEC foram comparados com FVC por meio dos coeficientes de correlação de Pearson ou Spearman, análise de desempenho diagnóstico e testes de concordância. A significância estatística foi estabelecida em p < 0,05.

**Resultados:**

Aumento da FVC foi observado em 96% dos pacientes (21,9 ± 5,5%), RT em 44% (1,3 ± 3%) e aumento do VEC em 33,3% (29,0 ± 4,0%). Correlações moderadas foram encontradas entre RT e FVC e entre VEC e FVC (r = 0,44 e r = 0,36, respectivamente; p = 0,001). RT e VEC demonstraram sensibilidades de 64,8% e 40,7%, especificidades de 77,8% e 74,1% e acurácias de 71% e 57%, respectivamente, utilizando FVC acima da mediana como referência para FM. A concordância entre FVC e VEC revelou uma diferença média de −0,167 e um valor de kappa de Cohen de 0,171.

**Conclusões:**

A RMC detecta FM em pacientes com valvopatia aórtica de grau importante confirmada por biópsia. RT e VEC apresentam correlação moderada com a FVC. A análise combinada de RT e VEC pode fornecer informações complementares; no entanto, estudos adicionais são necessários para determinar seu impacto prognóstico.

## Graphical abstract

**Figure:**
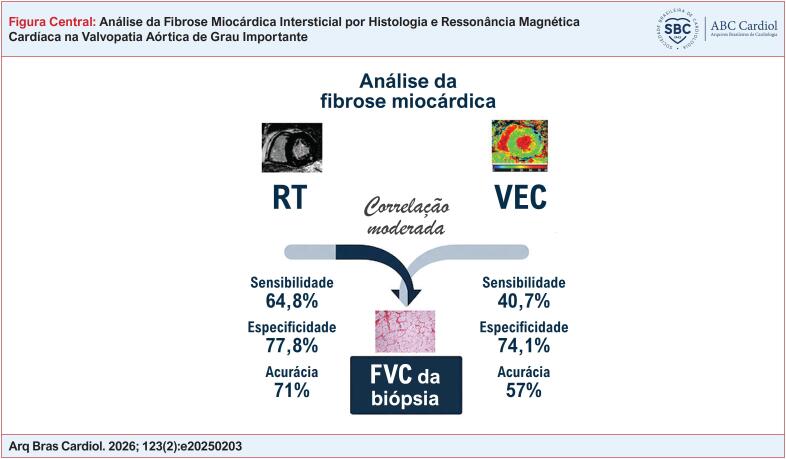
FVC: fração de volume de colágeno; RT: realce tardio; VEC: volume extracelular.

## Introdução

A valvopatia aórtica está associada a significativa morbidade e mortalidade, e sua prevalência tem aumentado devido ao envelhecimento populacional.^1-4^ Essa condição pode se apresentar como estenose aórtica (EAo) ou insuficiência aórtica (IAo)^1^ e resulta em sobrecarga hemodinâmica contínua do ventrículo esquerdo (VE). Essa sobrecarga desencadeia mecanismos adaptativos de hipertrofia e deposição progressiva de fibrose,^5-7^ que podem ser reversíveis em estágios iniciais,^8^ mas podem tornar-se irreversíveis em fases avançadas, particularmente devido à deposição de fibrose.^9^

A quantificação da fibrose pode ser realizada utilizando a fração de volume de colágeno (FVC), medida por coloração com Picrosirius red em amostras de biópsia miocárdica.^10,11^ Essa técnica é relativamente custo-efetiva e pode ser combinada com análise digital de imagens para melhorar a precisão da quantificação e reduzir a dependência do operador.^12^ No entanto, a biópsia miocárdica não é rotineiramente realizada na prática clínica para doenças valvares devido ao seu caráter invasivo, sendo, portanto, restrita a ambientes de pesquisa.

A ressonância magnética cardíaca (RMC) permite a quantificação da fibrose miocárdica (FM) regional por meio do realce tardio (RT). Na valvopatia aórtica, a presença de FM tem sido associada a pior prognóstico, conforme demonstrado por Debl et al.^13^ e Weidemann et al.^14^ Nigri et al.^15^ relataram boa acurácia da análise qualitativa do RT na valvopatia aórtica de grau importante quando comparada à histopatologia. Azevedo et al.^16^ demonstraram forte correlação entre a análise quantitativa do RT e a FVC (r = 0,69, p < 0,001).

A técnica do mapa T1 permite a detecção de fibrose intersticial e pode ser mais sensível que o RT para identificação precoce.^17-19^ O volume extracelular (VEC) miocárdico reflete a fração do tecido que não é composta por células.^20,21^ Flett et al.^22^ demonstraram sua correlação com achados histopatológicos em 18 pacientes com EAo. No entanto, um estudo de 2018 não encontrou associação entre VEC e FVC em 133 pacientes com EAo,^23^ embora tanto o RT quanto o VEC tenham sido associados a pior capacidade funcional.^22^

Devido ao número limitado de estudos avaliando FM, particularmente na IAo, este estudo teve como objetivo caracterizar a fibrose intersticial na valvopatia aórtica grave utilizando a FVC e compará-la com medidas derivadas da RMC, incluindo RT, T1 nativo e VEC.

## Métodos

Este subestudo incluiu pacientes recrutados em dois estudos observacionais prospectivos envolvendo indivíduos acompanhados na Unidade Clínica de Valvopatias do Instituto do Coração do Hospital das Clínicas da Faculdade de Medicina, Universidade de São Paulo. Ambos estudos foram aprovados pelo comitê de ética em pesquisa com seres humanos da instituição. Participaram pacientes de ambos os sexos, com idade entre 18 e 75 anos, portadores de valvopatia aórtica crônica de grau importante (predominantemente EAo ou IAo), independentemente da etiologia, e com indicação de tratamento cirúrgico valvar aórtico com base na Diretriz Brasileira de Valvopatias – SBC 2011/I Diretriz Interamericana de Valvopatias – SIAC 2011.^24^ Ambos os estudos originais já foram previamente publicados.^25,26^

Os pacientes foram recrutados entre junho de 2014 e setembro de 2019, após receberem informações completas sobre o protocolo do estudo e seus potenciais riscos e após assinarem o termo de consentimento livre e esclarecido. Os critérios de exclusão incluíram desistência do paciente em qualquer momento; doença valvar mitral associada maior que de grau leve; diabetes melito em uso de insulina; Clearance de creatinina estimada (calculada pela equação de Cockcroft-Gault) < 60 mL/min; claustrofobia que impedisse a realização da RMC; instabilidade hemodinâmica que impedisse a realização da RMC; fibrilação atrial ou outras arritmias cardíacas que prejudicassem a qualidade do mapa T1; e presença de dispositivos metálicos contraindicados para RMC ou que gerassem artefatos que limitassem a avaliação do mapa T1.

Os pacientes incluídos neste subestudo atenderam aos mesmos critérios de inclusão e exclusão dos estudos originais; no entanto, apenas aqueles com mapa T1 avaliável na RMC e amostras adequadas de biópsia miocárdica foram incluídos.

**Figura 1:**
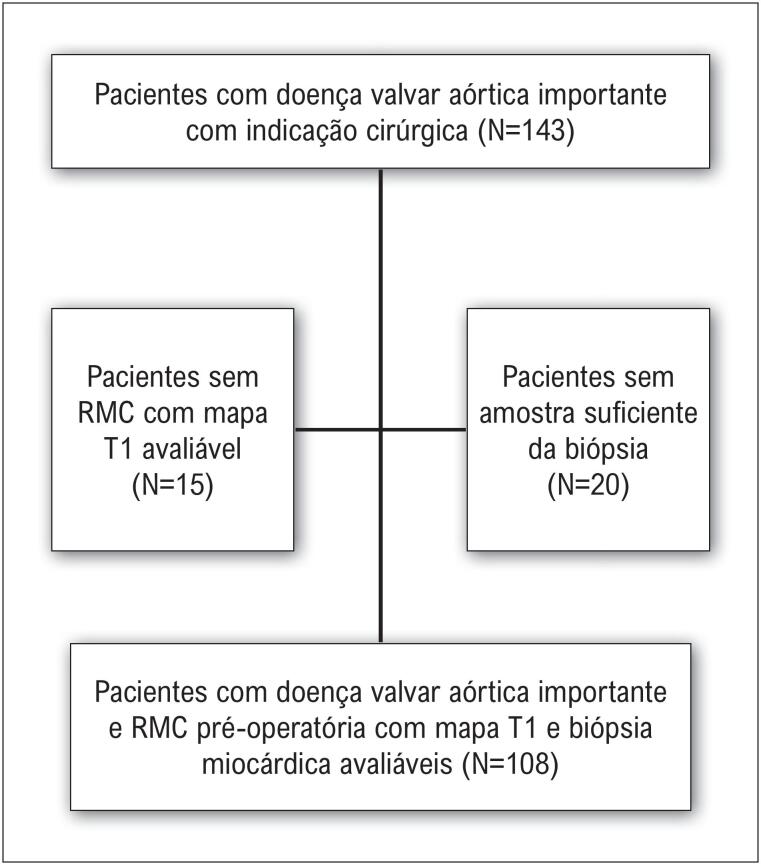
Fluxograma do estudo de seleção dos pacientes e critérios de inclusão. RMC: ressonância magnética cardíaca.

Entre os 143 pacientes incluídos nos 2 estudos primários, 15 não apresentaram mapas T1 com qualidade suficiente para análise, e 20 não possuíam amostras de biópsia avaliáveis devido a limitações intraoperatórias ou quantidade insuficiente de tecido para análise adequada. Isso resultou em uma amostra final de 108 pacientes com valvopatia aórtica de grau importante submetidos à RMC pré-operatória com mapa T1 e biópsia miocárdica avaliável (Figura 1).

### Ressonância magnética cardíaca: aquisição das imagens

As imagens foram adquiridas em um equipamento de 1,5 T (Achieva, Philips Healthcare, Amsterdã, Holanda), com os pacientes em posição supina, utilizando bobina de corpo e sincronização eletrocardiográfica retrospectiva. A RMC foi realizada no pré-operatório, até 90 dias antes da cirurgia.

Para avaliar a FM, as imagens de RT foram obtidas por meio de sequências gradiente-eco com pulsos preparatórios de inversão-recuperação^27,28^ e reconstruções sensíveis à fase (phase-sensitive inversion recovery - PSIR), com a finalidade de ajustar o tempo de inversão, a fim de anular o sinal do miocárdio normal (parâmetros utilizados: tempo de repetição: 6,1 ms; tempo de eco: 3,0 ms; flip angle 25°; resolução espacial: 1,5 x 2,0 x 8,0 mm). As imagens de RT foram adquiridas após um tempo mínimo de 10 minutos após a injeção do contraste à base de gadolínio intravenoso (Dotarem, Guerbet, Aulnay-Sous-Bois, França) na dose de 0,2 mmol/kg (0,4 mL/kg).

O T1 nativo (pré-contraste) e pós-contraste foi realizado utilizando sequência de pulso conhecida como MOLLI (modified inversion recovery Look-Locker)^18^ é baseada em método proposto por Look e Locker.^29^ Consiste em sequência de pulso de gradiente-eco com leitura em SSFP (Steady State Free Precession) e não segmentada do K-space (single-shot), precedida de pulsos de inversão-recuperação. O que permite a aquisição das imagens em apenas uma pausa respiratória. Este esquema de sequência MOLLI, conhecido como clássico é o mais utilizado na literatura, adquirido em pausa respiratória de 17 batimentos e gera 11 imagens a diferentes tempos do pulso de inversão-recuperação. Esta sequência utilizou o esquema conhecido com 3(3)3(3)5, que indicam 3 batimentos com aquisição seguidos de 3 batimentos de pausa, mais 3 batimentos de aquisição, seguidos de 3 batimentos de pausa, e finalmente 5 batimentos de aquisição. Os parâmetros utilizados foram: espessura de 10 mm, campo de visão 300 x 300 mm, matriz 152 x 150, flip angle 40º, tempo de inversão (TI) mínimo de 60 ms e incremento de TI 150 ms.

Para o mapa T1, imagens em eixo curto do VE foram adquiridas nas porções basal, medial e apical.

### Ressonância magnética cardíaca: análise das imagens

Todas as imagens foram armazenadas no formato *Digital Imaging Communications in Medicine* (DICOM) e analisadas com *software* comercialmente disponível (CVi42, versão 5.13.5; Circle Cardiovascular Imaging Inc., Calgary, Canadá).

O RT miocárdico foi quantificado por meio de uma técnica semiautomatizada baseada em limiar, definindo fibrose como intensidade de sinal > 5 desvios-padrão (DPs) acima do sinal médio do miocárdio remoto visualmente normal. O *software* forneceu a massa de RT (g), que também foi expressa como porcentagem da massa total do VE.

**Figura 2:**
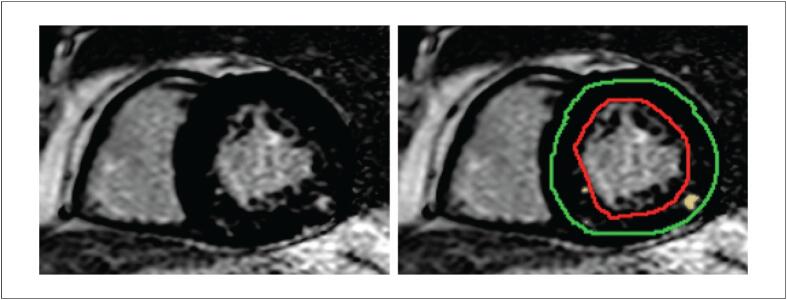
Quantificação do RT miocárdico. Imagens em eixo curto mostrando a mensuração do RT em um participante representativo do estudo. RT: realce tardio.

A análise de T1 foi realizada delineando-se uma região de interesse no miocárdio com base nos contornos endocárdico e epicárdico, excluindo estruturas extracardíacas e a cavidade ventricular (Figura 2). As medidas foram obtidas para cada segmento das porções basal, medial e apical. Os valores de T1 foram calculados utilizando um modelo exponencial e corrigidos para a frequência cardíaca. A partir dos valores obtidos, o VEC miocárdico foi calculado de acordo com a seguinte fórmula:^20^

$$\text{VEC}=(1-\text{hematócrito})\times \frac{{\left(1/\text{T}1\right)}_{\text{mio-pós}}-{\left(1/\text{T}1\right)}_{\text{mio-pré}}}{{\left(1/\text{T}1\right)}_{\text{sg-pós}}-{\left(1/\text{T}1\right)}_{\text{sg-pré}}}$$

VEC: Volume extracelular do miocárdio (volume de distribuição extracelular do contraste do miocárdio)

(1/T1)_mio-pós_: inverso do T1 miocárdio após injeção de contraste

(1/T1)_mio-pré_: inverso do T1 miocárdio antes da injeção de contraste

(1/T1)_sg-pós_: inverso do T1 sanguíneo após injeção de contraste

(1/T1)_sg-pré_: inverso do T1 sanguíneo antes da injeção de contraste

O VEC representa a fração do tecido miocárdico composta pelo espaço extracelular e, portanto, reflete a FM difusa/intersticial. No mesmo dia da RMC, foi obtida uma amostra de sangue periférico para análise de hemograma completo; o hematócrito foi utilizado na fórmula.

**Figura 3:**
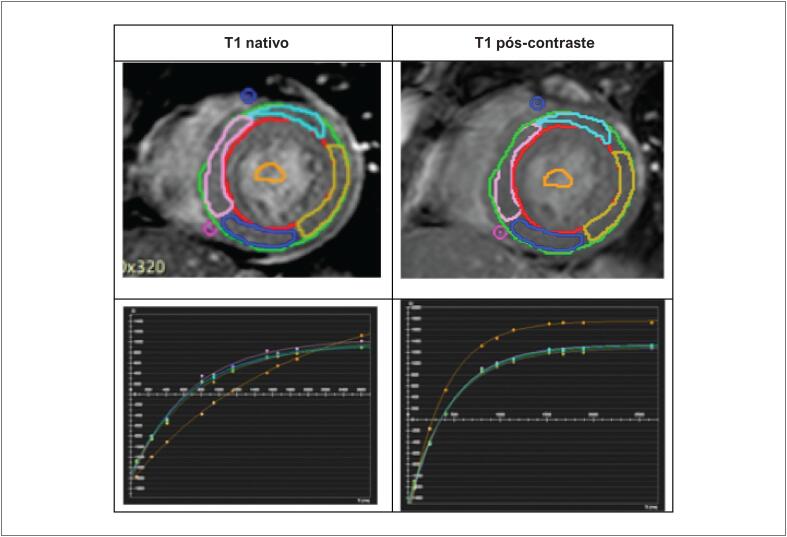
Medidas segmentares de T1 nativo e pós-contraste e suas respectivas curvas de relaxamento. Cortes em eixo curto cardíaco e curvas de relaxamento de T1 nativo em um participante representativo do estudo.

**Figura 4:**
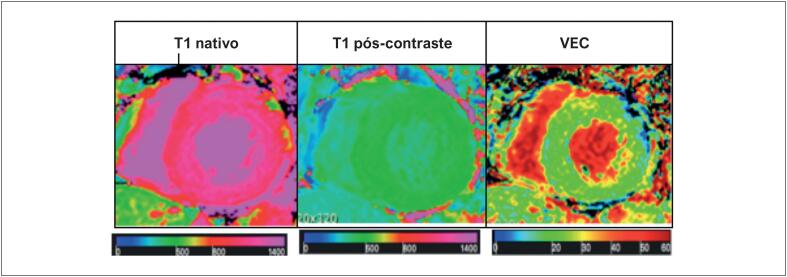
Imagens de mapa T1 e VEC em um participante representativo do estudo. Cortes em eixo curto cardíaco ilustrando uma escala de cores graduada correspondente aos valores locais medidos. VEC: volume extracelular.

O T1 nativo e o VEC foram quantificados em regiões miocárdicas sem RT, e os valores globais foram calculados como a média dos segmentos miocárdicos analisados (Figura 3; Figura 4).

### Biópsia endomiocárdica

Durante o tratamento cirúrgico da valvopatia aórtica, foi obtido um fragmento endomiocárdico pesando entre 25 e 50 mg do septo interventricular basal, abaixo da comissura entre os folhetos coronarianos esquerdo e direito. O fragmento foi fixado em formalina tamponada a 10% por 24 horas e enviado ao laboratório institucional de patologia para processamento rotineiro e inclusão em parafina.

**Figura 5:**
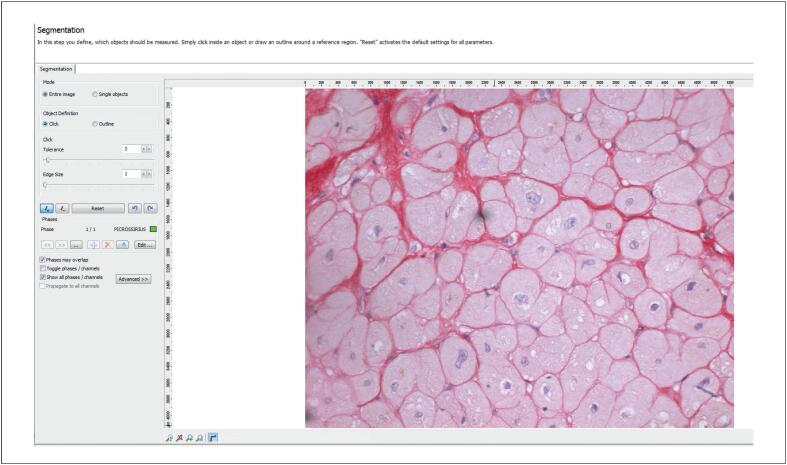
Etapa inicial da quantificação da fibrose intersticial na histologia. Campo corado com Picrosirius red antes da quantificação automatizada. A fibrose intersticial aparece em vermelho escuro; os miócitos aparecem pálidos com núcleos azulados. O observador seleciona o tom vermelho a ser quantificado como fibrose.

**Figura 6:**
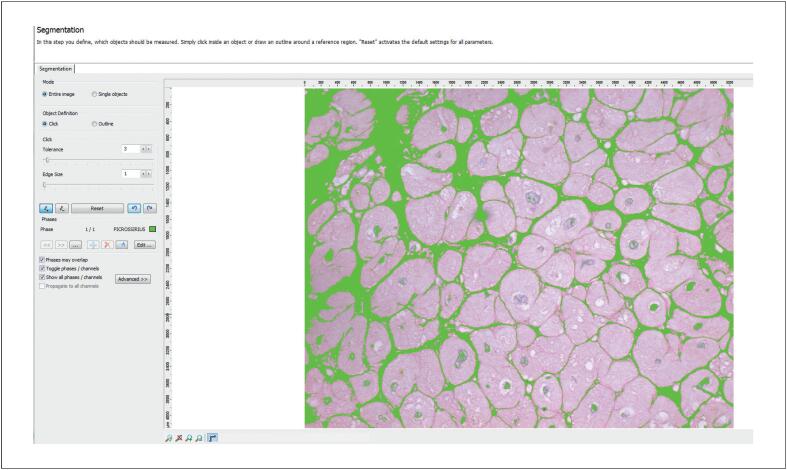
Quantificação da fibrose intersticial na histologia. O mesmo campo mostrado na Figura 5 após a detecção automatizada, com as áreas de colágeno destacadas em verde. Ajustes finos manuais foram realizados quando necessário.

Cortes com 5 *µ*m de espessura foram realizados e corados com hematoxilina e eosina para avaliação histológica padrão e com Picrosirius red para avaliação e quantificação da fibrose intersticial. O Picrosirius red cora colágeno tipo I e tipo III em tons de vermelho. As imagens histológicas foram capturadas utilizando uma câmera AxioCam HR (Carl Zeiss, Alemanha) acoplada a um microscópio Axio Imager A1 (Carl Zeiss, Alemanha), integrados ao software AxioVision (versão 4.6; Carl Zeiss, Alemanha) (Figura 5; Figura 6).

As imagens foram adquiridas aleatoriamente com aumento de 400×, com um mínimo de 10 e um máximo de 20 campos por lâmina. Regiões contendo vasos > 50 *µ*mde diâmetro e áreas com > 50% de fibrose foram excluídas para evitar fibrose consolidada e regiões com grande retração tecidual. O endocárdio não foi analisado. A avaliação quantitativa da fibrose intersticial foi realizada pelo cálculo da FVC miocárdica por histomorfometria computadorizada, expressa como a porcentagem da área de colágeno intersticial em relação à área total de miocárdio analisada em cada amostra.

### Análise estatística

As estatísticas descritivas são apresentadas como frequências absolutas e relativas para variáveis categóricas. Variáveis contínuas são apresentadas como média ± DP quando normalmente distribuídas e como mediana (intervalo interquartil [IIQ]) quando não normalmente distribuídas.

A normalidade das variáveis contínuas foi avaliada pelo teste de Shapiro–Wilk, e os testes estatísticos subsequentes foram selecionados de acordo. As associações entre as métricas de fibrose derivadas da RMC (medidas baseadas em mapa T1 e RT) e a histopatologia foram avaliadas por regressão linear e pelos coeficientes de correlação de Pearson ou Spearman, conforme apropriado. Os pressupostos da regressão linear foram avaliados e, embora o teste estatístico tenha indicado violação da normalidade dos resíduos em algumas comparações, as análises visuais não apontaram desvios relevantes. Considerando o tamanho da amostra, o modelo foi utilizado para análise, e os resultados interpretados com a devida cautela.

O desempenho diagnóstico da RMC para detecção de fibrose histológica foi avaliado por meio de curva característica de operação do receptor (ROC, em inglês), com cálculo da área sob a curva (AUC, em inglês) e intervalos de confiança de 95% (ICs 95%). A concordância entre os métodos de detecção de fibrose foi avaliada por análise de Bland–Altman e, quando aplicável, pelo coeficiente kappa de Cohen.

Todas as análises foram realizadas utilizando o Stata/SE 13 (StataCorp LP, College Station, TX, EUA). Todos os testes foram bicaudais, e a significância estatística foi estabelecida em p < 0,05.

## Resultados

### Características clínicas e demográficas

Dos 108 indivíduos com valvopatia aórtica de grau importante, 28 (26%) apresentavam IAo. A mediana de idade foi de 63 anos, e 65% dos participantes eram do sexo masculino (Tabela 1).

**Tabela 1:** Características demográficas e clínicas pré-operatórias (n = 108)

Variável	Valor
Idade, anos	63 (56–70)
Sexo masculino	70 (65%)
Creatinina, mg/dl	1,08 ± 0,3
Diabetes melito	24 (22%)
Hipertensão arterial sistêmica	71 (66%)
Doença arterial coronariana	2 (2%)
Dislipidemia	40 (37%)

Os dados são apresentados como média ± desvio padrão para variáveis contínuas com distribuição normal, mediana (intervalo interquartil) para variáveis com distribuição não normal e valores absolutos e percentuais para variáveis categóricas.

### Avaliação histológica da fração de volume de colágeno

A FVC miocárdica média na amostra do estudo foi de 21,9 ± 5,5% (Tabela 2). A FM intersticial foi considerada patologicamente aumentada quando a FVC excedia a média mais 2 DPs dos valores observados em um grupo controle, correspondendo a um valor de referência de 9,6%, conforme previamente estabelecido.^16^ No entanto, como os valores médios de FVC nesta coorte foram marcadamente superiores a esse valor de referência, a mediana da FVC foi adotada como ponto de corte para fibrose anormal (≥ 22,25%), resultando em 54 pacientes classificados com FVC aumentada. Esse limiar é consistente com valores clinicamente relevantes relatados em pesquisas anteriores.^30,31^

**Tabela 2:** Parâmetros de avaliação da fibrose por ressonância magnética cardíaca pré-operatória e histologia

Parâmetro	Média/mediana
RT (%)	0 (0–1,74)
VEC global sem RT (%)	29 ± 4,20
FVC (%)	22 ± 5,50

Os dados são apresentados como média ± desvio padrão para variáveis contínuas com distribuição normal e mediana (intervalo interquartil) para variáveis com distribuição não normal. FVC: fração de volume de colágeno; RT: realce tardio; VEC: volume extracelular.

Utilizando o valor de referência de 9,6%, 96% dos pacientes (n = 104) da amostra total apresentaram FVC aumentada.

### Fibrose miocárdica macroscópica

A FM regional detectada pelo RT esteve presente em 44% dos pacientes (n = 47). A mediana da porcentagem de RT em relação à massa total do VE foi 0 (IIQ, 0–1,74), com valor médio de 1,3% na amostra global (Tabela 2).

### Avaliação do volume extracelular: fibrose intersticial

Os valores médios de VEC medidos em regiões miocárdicas sem RT foram de 29 ± 4% (Tabela 2). Utilizando um valor de referência de 30%, 32% dos pacientes (n = 35) apresentaram VEC elevado.

### Correlações entre as medidas de fibrose derivadas da ressonância magnética cardíaca e a fração de volume de colágeno

**Figura 7:**
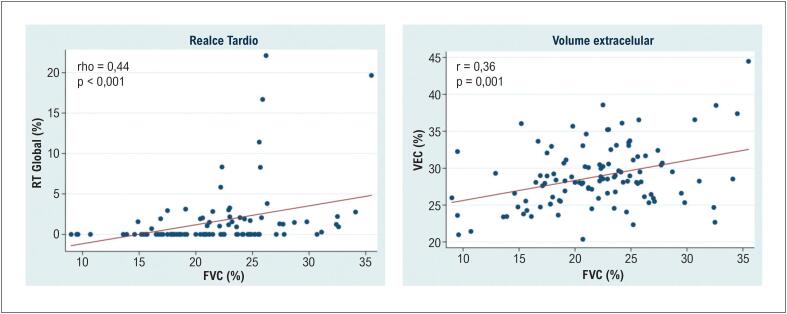
Regressão linear entre RT e FVC. FVC: fração de volume de colágeno; VEC: volume extracelular.

Devido à distribuição assimétrica dos valores de RT e à presença de outliers identificados na análise exploratória, utilizou-se a correlação de Spearman para avaliar a associação entre RT e FVC. Observou-se uma correlação positiva moderada e estatisticamente significativa entre RT e FVC (ρ = 0,44; p < 0,001), indicando uma associação monotônica. Também foi observada correlação moderada entre VEC e FVC (r = 0,36; p = 0,001) (Tabela 3; Figura 7).

**Tabela 3:** Correlação entre os parâmetros de fibrose derivados da ressonância magnética cardíaca e a fração de volume de colágeno

Parâmetro	ρ/r	Valor p
RT global (%)	0,44	< 0,001
VEC global sem RT (%)	0,36	0,001

FVC: fração de volume de colágeno; RT: realce tardio; VEC: volume extracelular.

### Desempenho diagnóstico da ressonância magnética cardíaca

**Figura 8:**
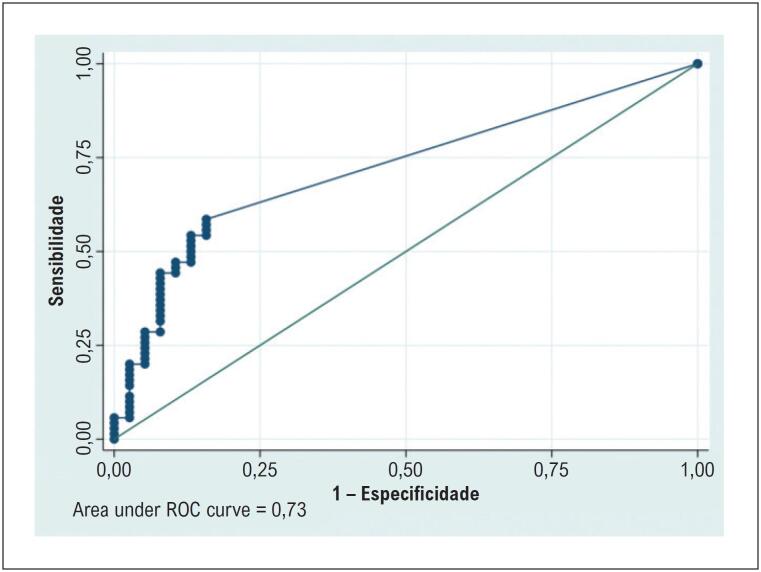
Curva ROC do RT para predição de fibrose miocárdica por biópsia. FVC: fração de volume de colágeno; ROC: característica de operação do receptor; RT: realce tardio.

A sensibilidade do RT para detectar FM confirmada histologicamente foi de 64,8%, com especificidade de 77,8%, acurácia de 71% e AUC de 0,73 (Tabela 4; Figura 8).

**Tabela 4:** Desempenho diagnóstico do realce tardio para detecção de fibrose histológica

Parâmetro	Estimativa (%)	IC 95%
Sensibilidade	64,80	50,60–77,30
Especificidade	77,80	64,40–88,00
Acurácia	71,00	62,00–80,00
AUC	0,73	0,68–0,78

AUC: área sob a curva característica de operação do receptor; IC: intervalo de confiança.

**Figura 9:**
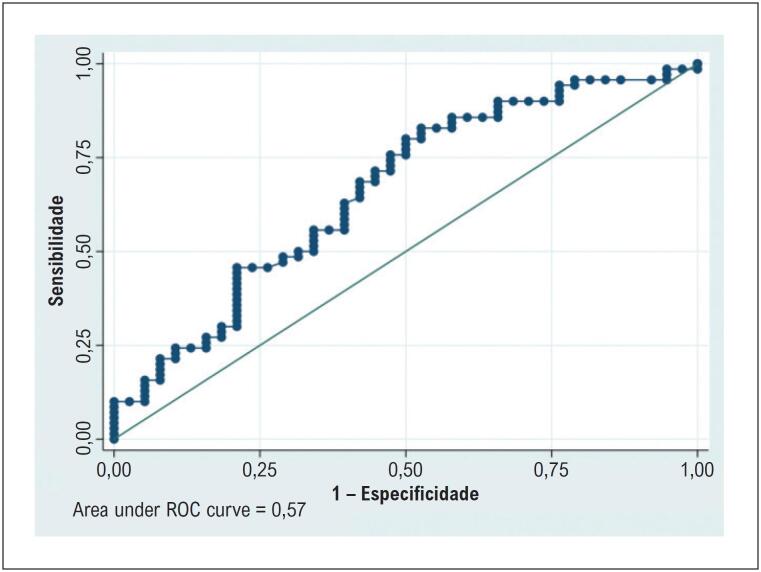
Curva característica de operação do receptor (ROC) comparando FVC e VEC. AUC: área sob a curva ROC; FVC: fração de volume de colágeno; VEC: volume extracelular.

**Figura 10:**
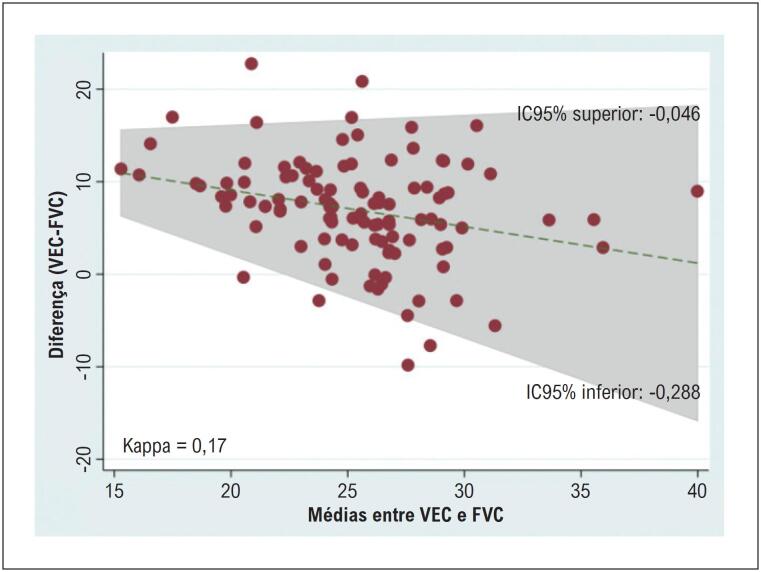
Análise de concordância de Bland–Altman entre FVC e VEC. FVC: fração de volume de colágeno; IC: intervalo de confiança; VEC: volume extracelular.

O desempenho diagnóstico do VEC para identificar FVC aumentada (≥ 22,25%) apresentou sensibilidade de 40,7%, especificidade de 74,1%, acurácia de 57% e AUC de 0,574. A análise de concordância pelo método de Bland–Altman demonstrou viés médio de −0,167 (IC 95%, −0,288 a −0,046). Os limites de concordância exibiram um padrão trapezoidal, indicando viés proporcional e aumento da variabilidade com valores médios mais elevados. O coeficiente kappa de Cohen foi baixo (0,171), indicando concordância muito fraca entre o VEC e as medidas histológicas de fibrose. Esses achados destacam limitações importantes na concordância que devem ser consideradas na interpretação dos resultados do VEC (Tabela 5; Figura 9; Figura 10).

**Tabela 5:** Análise da curva característica de operação do receptor (ROC) do volume extracelular para predição de fibrose miocárdica histológica

Parâmetro	Estimativa (%)	IC 95%
Sensibilidade	41	28–55
Especificidade	74	60,30–85
Acurácia	57	48–67
AUC	0,57	0,49–0,66

AUC: área sob a curva ROC; IC: intervalo de confiança.

## Discussão

Os achados deste estudo, resumidos na Figura Central, são consistentes com pesquisas anteriores ao demonstrar uma carga substancial de FM em pacientes com valvopatia aórtica de grau importante. No entanto, uma limitação importante refere-se ao local de coleta da biópsia. A obtenção de tecido foi restrita ao septo interventricular, resultando em uma amostra limitada que pode ter restringido a avaliação histopatológica, considerando que apenas um único fragmento miocárdico foi obtido durante a cirurgia. Estudos prévios relataram correlações mais fortes entre VEC e histologia quando múltiplos (n = 16) blocos de tecido de diferentes segmentos miocárdicos foram analisados, como em investigações com corações explantados de pacientes com cardiomiopatia dilatada ou isquêmica submetidos a transplante cardíaco.^32^ Além disso, a FM na valvopatia aórtica não se limita ao septo interventricular, o que pode reduzir ainda mais a representatividade de uma única biópsia septal.^16^

A média da FVC observada nesta coorte (22 ± 5,5%) reflete um estágio avançado de remodelamento intersticial. Esse achado é consistente com estudos anteriores que indicam que a sobrecarga hemodinâmica crônica desempenha papel central na ativação de mecanismos de hipertrofia e na deposição de matriz extracelular.^10,11^

A quantificação da fibrose por RMC mostrou correlações moderadas entre RT e FVC (r = 0,44; p < 0,001), bem como entre VEC e FVC (r = 0,36; p = 0,001). Esses resultados são direcionalmente consistentes com os relatados por Azevedo et al.,^16^ que demonstraram forte correlação entre RT e FVC (r = 0,69; p < 0,001). A menor magnitude de correlação observada no presente estudo pode ser explicada por diferenças metodológicas na quantificação do RT e pela maior proporção de pacientes com EAo incluídos nesta coorte.

A FM macroscópica detectada pelo RT esteve presente em 44% dos pacientes, sustentando o conceito de que a deposição de colágeno em estágios avançados da valvopatia aórtica é heterogênea e regionalmente distribuída, conforme descrito por Nigri et al.^15^ No entanto, o RT apresentou sensibilidade de 64,8%, especificidade de 77,8%, acurácia global de 71% e AUC de 0,73 para a detecção de fibrose histológica. Esses achados indicam que, embora o RT seja uma ferramenta valiosa para identificar FM, pode subestimar a carga total de fibrose intersticial, particularmente em pacientes sem extensa deposição focal de colágeno.

O VEC apresentou correlação moderada com a FVC, em contraste com um estudo de 2018 que não encontrou associação entre VEC e FVC, possivelmente porque aquela coorte era composta exclusivamente por pacientes com EAo, ao contrário da população do presente estudo. Ainda assim, o VEC demonstrou desempenho diagnóstico inferior ao do RT (AUC = 0,57), com sensibilidade de 40,7% e especificidade de 74,1%. A sensibilidade reduzida do VEC pode ser parcialmente atribuída à heterogeneidade da fibrose, uma vez que essa técnica quantifica a expansão do espaço extracelular sem distinguir diretamente o colágeno de outros componentes intersticiais. A análise de Bland–Altman revelou um viés médio de −0,167 (IC 95%, −0,288 a −0,046), indicando leve subestimação da fibrose pelo VEC. A análise de concordância apresentou baixo valor de kappa de Cohen (0,171), indicando concordância apenas fraca entre VEC e FVC.

De modo geral, os parâmetros derivados do mapa T1 destacam o papel da RMC não apenas como modalidade diagnóstica não invasiva, mas também como ferramenta para ampliar a compreensão da fisiopatologia da valvopatia aórtica, particularmente dos mecanismos de remodelamento miocárdico e formação de fibrose. As correlações moderadas observadas entre os parâmetros derivados da RMC e a FVC sustentam sua aplicabilidade na caracterização da FM e potencialmente na avaliação longitudinal. Estudos recentes também demonstraram associações entre VEC e desfechos prognósticos pós-operatórios em pacientes com valvopatia aórtica, especialmente quando o VEC é indexado à massa do VE, o que reforça a relevância clínica potencial dessas medidas.^23^

## Conclusão

A FVC encontra-se marcadamente aumentada na valvopatia aórtica de grau importante, apesar da limitação amostral inerente à coleta por biópsia restrita ao septo interventricular. Esses achados destacam a moderada especificidade, mas baixa sensibilidade do VEC para a detecção da fibrose miocárdica, indicando que, embora o VEC possa auxiliar na exclusão de fibrose avançada, seu uso como marcador diagnóstico isolado para fibrose intersticial permanece limitado.

De modo geral, nossos resultados reforçam que a fibrose miocárdica é um processo multifatorial e que as técnicas baseadas em imagem apresentam limitações inerentes na sua detecção e quantificação. A avaliação combinada utilizando RT e VEC pode fornecer informações complementares, particularmente para a identificação precoce da fibrose intersticial. Estudos adicionais são necessários para determinar o impacto prognóstico dessas medidas na evolução clínica da valvopatia aórtica e para avaliar o potencial do mapa T1 como marcador mais sensível de fibrose miocárdica precoce. Este estudo ressalta a relevância da RMC na avaliação da fibrose e da microestrutura miocárdicas como fator no entendimento do remodelamento ventricular e com potencial impacto na escolha terapêutica e seu melhor momento.

## Data Availability

Os conteúdos subjacentes ao texto da pesquisa estão contidos no manuscrito.
