# Supplementary material for: Análise da Fibrose Miocárdica Intersticial por Histologia e Ressonância Magnética Cardíaca na Valvopatia Aórtica de Grau Importante
Source: Arq Bras Cardiol. 2026 Apr 16;123(2):e20250203. [Article in Portuguese] doi: 10.36660/abc.20250203 (PMC13148856; doi:10.36660/abc.20250203)
Supplement: Translation

# Translation: Interstitial Myocardial Fibrosis Analysis by Histology and Cardiac Magnetic Resonance in Severe Aortic Valve Disease

**DOI:** 10.36660/abc.20250203i

**Authors:** Juliana Hiromi Silva Matsumoto Bello, Lea M.M.F. Demarchi, Rafael Almeida Fonseca, Gabriele N. Lima, Lucas José Tachotti Pires, Vitor E. E. Rosa, Roney Orismar Sampaio, Paulo S. Gutierrez, Vera D. Aiello, Flávio Tarasoutchi, Carlos E. Rochitte

**Affiliations:** 1 São Paulo SP Brazil Instituto do Coração do Hospital das Clínicas da Faculdade de Medicina da Universidade de São Paulo, São Paulo, SP – Brazil; 2 São Paulo SP Brazil Hospital das Clínicas da Faculdade de Medicinada Universidade de São Paulo, São Paulo, SP – Brazil

**Keywords:** Aortic Valve Disease, Miocardio, Fibrosis, Magnetic Resonance Imaging, Histology

## Abstract

**Background:**

Aortic valve disease may lead to the accumulation of myocardial fibrosis (MF). Cardiac magnetic resonance (CMR) with late gadolinium enhancement (LGE) evaluates regional fibrosis but does not detect interstitial fibrosis, which can be estimated by extracellular volume (ECV) using native (pre-contrast) and post-contrast T1 mapping.

**Objectives:**

To compare MF measured by collagen volume fraction (CVF) in myocardial biopsy with LGE and ECV in patients with severe aortic valve disease.

**Methods:**

A total of 108 patients undergoing aortic valve replacement with myocardial biopsy and preoperative CMR were included. LGE and ECV were compared with CVF using Pearson or Spearman correlation coefficients, diagnostic performance analysis, and agreement testing. Statistical significance was set at p < 0.05.

**Results:**

Increased CVF was observed in 96% of patients (21.9 ± 5.5%), LGE in 44% (1.3 ± 3%), and increased ECV in 33.3% (29.0 ± 4.0%). Moderate correlations were found between LGE and CVF and between ECV and CVF (r = 0.44 and r = 0.36, respectively; p = 0.001). LGE and ECV demonstrated sensitivities of 64.8% and 40.7%, specificities of 77.8% and 74.1%, and accuracies of 71% and 57%, respectively, using CVF above the median as the reference for MF. Agreement between CVF and ECV revealed a mean difference of −0.167 and a Cohen's kappa value of 0.171.

**Conclusions:**

CMR detects MF in patients with severe aortic valve disease confirmed by biopsy. LGE and ECV show moderate correlation with CVF. Combined analysis of LGE and ECV may provide complementary information; however, additional studies are needed to determine their prognostic impact.

## Introduction

Aortic valve disease is associated with significant morbidity and mortality, and its prevalence has increased due to population aging.^1-4^ This condition may present as aortic stenosis (AS) or aortic regurgitation (AR)^1^ and results in continuous hemodynamic overload of the left ventricle (LV). This overload triggers adaptive hypertrophic mechanisms and progressive fibrotic deposition,^5-7^ which may be reversible in earlier stages^8^ but can become irreversible in advanced phases, particularly due to interstitial fibrosis deposition.^9^

Fibrosis quantification can be performed using collagen volume fraction (CVF) measured by Picrosirius red staining in myocardial biopsy samples.^10,11^ This technique is relatively cost-effective and can be combined with digital image analysis to improve quantification accuracy and reduce operator dependency.^12^ However, myocardial biopsy is not routinely performed in clinical practice for valvular disease due to its invasive nature and is therefore mainly restricted to research settings.

Cardiac magnetic resonance (CMR) enables quantification of regional myocardial fibrosis (MF) through late gadolinium enhancement (LGE). In aortic valve disease, the presence of MF has been associated with worse prognosis, as demonstrated in studies by Debl et al.^13^ and by Weidemann et al.^14^ Nigri et al.^15^ reported good accuracy of qualitative LGE analysis in severe aortic valve disease when compared with histopathology. Azevedo et al.^16^ demonstrated a strong correlation between quantitative LGE analysis and CVF(r = 0.69, p < 0.001).

T1 mapping allows detection of interstitial fibrosis and may be more sensitive than LGE for early identification.^17-19^ Myocardial extracellular volume (ECV) reflects the tissue fraction that is not composed of cells.^20,21^ Flett et al.^22^ demonstrated its correlation with histopathological findings in 18 patients with AS. However, a 2018 study found no association between ECV and CVF in 133 patients with AS,^23^ although both LGE and ECV were associated with worse functional capacity.^24^

Because of the limited number of studies evaluating MF, particularly in AR, this study aimed to characterize interstitial fibrosis in severe aortic valve disease using CVF and to compare it with CMR-derived measures, including LGE, native T1 mapping, and ECV.

## Methods

This sub-study included patients enrolled in two prospective observational studies involving individuals followed at the Valvular Heart Disease Clinical Unit of the Heart Institute of the Hospital das Clínicas of the Universidade de São Paulo Medical School. Both studies were approved by the institutional human research ethics committee. Participants included patients aged between 18 and 75 years of either sex with chronic severe aortic valve disease (predominantly AS or AR), regardless of etiology, and with an indication for surgical aortic valve treatment based on the Brazilian Guidelines for Heart Valve Disease – 2011/I Inter-American Guideline for Heart Valve Disease – 2011 SIAC.^25^ Both original studies have been previously published.^23,26^

Patients were recruited between June 2014 and September 2019 after receiving full information about the study protocol and its potential risks and after providing written informed consent. Exclusion criteria included patient withdrawal at any time; associated mitral valve disease greater than mild severity; insulin-treated diabetes mellitus; estimated creatinine clearance (calculated using the Cockcroft-Gault equation) < 60 mL/min; claustrophobia preventing CMR; hemodynamic instability preventing CMR; atrial fibrillation or other cardiac arrhythmias that impaired T1 mapping quality; and the presence of metallic devices contraindicating CMR or causing artifacts that limited T1 map evaluation.

Patients included in this sub-study met the same inclusion and exclusion criteria as the original studies; however, only those with evaluable T1 mapping on CMR and adequate myocardial biopsy samples were included.

Among the 143 patients enrolled in the 2 primary studies, 15 did not have T1 maps of sufficient quality for analysis, and 20 did not have evaluable biopsy samples due to intraoperative limitations or insufficient tissue for adequate analysis. This resulted in a final sample of 108 patients with severe aortic valve disease who underwent preoperative CMR with analyzable T1 mapping and evaluable myocardial biopsy (Figure 1).

**Figure 1:** Study flowchart of patient selection and inclusion criteria. CMR: cardiac magnetic resonance.

### Cardiac magnetic resonance: image acquisition

Images were acquired on a 1.5-T scanner (Achieva, Philips Healthcare, Amsterdam, the Netherlands) with patients in the supine position, using a body coil and retrospective electrocardiographic gating. CMR was performed preoperatively, up to 90 days before surgery.

To assess myocardial fibrosis, LGE imaging was obtained using gradient-echo sequences with inversion-recovery preparation pulses^27,28^ and phase-sensitive inversion recovery reconstruction, allowing adjustment of the inversion time to null signal from normal myocardium (repetition time, 6.1 ms; echo time, 3.0 ms; flip angle, 25°; spatial resolution, 1.5 × 2.0 × 8.0 mm). LGE images were acquired at least 10 minutes after intravenous administration of a gadolinium-based contrast agent (Dotarem, Guerbet, Aulnay-Sous-Bois, France) at a dose of 0.2 mmol/kg (0.4 mL/kg).

Native (pre-contrast) and post-contrast T1 mapping were performed using the modified Look-Locker inversion recovery (MOLLI) sequence,^18^ based on the method originally described by Look and Locker.^29^ This approach uses a gradient-echo readout with single-shot, non-segmented k-space acquisition under steady-state free precession, preceded by inversion-recovery pulses, enabling image acquisition within a single breath-hold. The MOLLI scheme used in this study was the classic 3(3)3(3)5 protocol, acquired over a 17–heartbeat breath-hold and yielding 11 images at different inversion times. This scheme consists of 3 beats with image acquisition followed by 3 recovery beats, then another 3 acquisition beats followed by 3 recovery beats, and finally 5 acquisition beats. Imaging parameters were as follows: slice thickness, 10 mm; field of view, 300 × 300 mm; matrix, 152 × 150; flip angle, 40°; minimum inversion time, 60 ms; inversion-time increment, 150 ms.

For T1 mapping, short-axis images of the LV were acquired at basal, mid-ventricular, and apical levels.

### Cardiac magnetic resonance: image analysis

All images were stored in Digital Imaging Communications in Medicine format and analyzed using commercially available software (CVi42, version 5.13.5; Circle Cardiovascular Imaging Inc, Calgary, Canada).

Myocardial LGE was quantified using a semi-automated threshold-based technique, defining fibrosis as signal intensity > 5 standard deviations (SDs) above the mean signal of remote, visually normal myocardium. The software provided LGE mass (g), which was also expressed as a percentage of total LV mass.

T1 analysis was performed by delineating a region of interest in the myocardium based on endocardial and epicardial contours, while excluding extracardiac structures and the ventricular cavity (Figure 2). Measurements were obtained for each segment at basal, mid-ventricular, and apical levels. T1 values were derived using an exponential model and corrected for heart rate. Using the resulting T1 values, myocardial ECV was calculated according to the following formula:^20^

**Figure 2:** Quantification of myocardial LGE. Short-axis images showing LGE measurement in a representative study participant. LGE: late gadolinium enhancement.

$$\text{ECV}=(1-\text{hematocrit})\text{X}\frac{{\left(1/\text{T}1\right)}_{\text{post-myocardial}}-{\left(1/\text{T}1\right)}_{\text{pre-myocardial}}}{{\left(1/\text{T}1\right)}_{\text{post-blood}}-{\left(1/\text{T}1\right)}_{\text{pre-blood}}}$$

ECV: Extracellular myocardial volume (extracellular distribution volume of contrast in the myocardium)

(1/T1)_post-myocardial_: inverse of myocardial T1 after contrast injection

(1/T1)_pre-myocardial_: inverse of myocardial T1 before contrast injection

(1/T1)_post-blood_: inverse of blood T1 after contrast injection

(1/T1)_pre-blood_: inverse of blood T1 before contrast injection

ECV represents the fraction of myocardial tissue composed of extracellular space and therefore reflects diffuse/interstitial myocardial fibrosis. On the same day as CMR, a peripheral blood sample was obtained for complete blood count analysis; hematocrit was used in Equation (1).

Native T1 and ECV were quantified in myocardial regions without LGE, and global values were calculated as the mean of the analyzed myocardial segments (Figure 3; Figure 4)).

**Figure 3:** Segmental native and post-contrast T1 measurements and corresponding relaxation curves. Short-axis views and native T1 relaxation curves in a representative study participant.

**Figure 4:** T1 mapping and ECV images in a representative study participant. Short-axis views illustrating a graded color scale corresponding to the local measured values. ECV: extracellular volume.

### Endomyocardial biopsy

During surgical treatment of aortic valve disease, an endomyocardial specimen weighing 25–50 mg was obtained from the basal interventricular septum, below the commissure between the left and right coronary cusps. The specimen was fixed in 10% buffered formalin for 24 hours and sent to the institutional pathology laboratory for routine processing and paraffin embedding.

Sections 5 *µ*m thick were cut and stained with hematoxylin and eosin for standard histological assessment and with Picrosirius red for evaluation and quantification of interstitial fibrosis. Picrosirius red stains type I and type III collagen in red hues. Histological images were captured using an AxioCam HR camera (Carl Zeiss, Germany) coupled to an Axio Imager A1 microscope (Carl Zeiss, Germany), integrated with AxioVision software (version 4.6; Carl Zeiss, Germany) (Figures 5; Figure 6).

**Figure 5:** Initial step of interstitial fibrosis quantification in histology. Picrosirius red–stained field before automated quantification. Interstitial fibrosis appears as dark red; myocytes appear pale with bluish nuclei. The observer selects the red hue to be quantified as fibrosis.

**Figure 6:** Interstitial fibrosis quantification in histology. The same field shown in Figure 5 after automated detection, with collagen areas highlighted in green. Manual fine adjustments were performed when needed.

Images were acquired randomly at 400× magnification, with a minimum of 10 and a maximum of 20 fields per slide. Regions containing vessels > 50 *µ*m in diameter and areas with > 50% fibrosis were excluded to avoid consolidated scar and regions with prominent tissue retraction artifacts. The endocardium was not analyzed. Quantitative assessment of interstitial fibrosis was performed by calculating myocardial CVF using computerized histomorphometry, expressed as the percentage of interstitial collagen area relative to the total myocardial area analyzed in each sample.

### Statistical analysis

Descriptive statistics are presented as absolute and relative frequencies for categorical variables. Continuous variables are reported as mean ± SD when normally distributed and as median (interquartile range [IQR]) when non-normally distributed.

Normality of continuous variables was assessed using the Shapiro–Wilk test, and subsequent statistical testing was selected accordingly. Associations between CMR-derived fibrosis metrics (T1 mapping–based measures and LGE) and histopathology were evaluated using linear regression and Pearson or Spearman correlation coefficients, as appropriate. Assumptions for linear regression were examined. Although statistical testing indicated non-normality of residuals in some comparisons, visual inspection did not suggest meaningful deviations. Because of the sample size, the regression model was retained and results were interpreted with appropriate caution.

The diagnostic performance of CMR for detection of histological fibrosis was assessed using receiver operating characteristic (ROC) curves, with calculation of the area under the curve (AUC) and 95% CIs. Agreement between fibrosis detection methods was evaluated using Bland–Altman analysis and, when applicable, Cohen's kappa.

All analyses were performed using Stata/SE 13 (StataCorp LP, College Station, TX, USA). All tests were two-sided, and statistical significance was set at p < 0.05. Additional analyses were conducted within predefined subgroups.

## Results

### Clinical and demographic characteristics

Of the 108 individuals with severe aortic valve disease, 28 (26%) had AR. The median age was 63 years (range 56–70), and 65% of participants were male (Table 1).

**Table 1:** Preoperative demographic and clinical characteristics (n = 108)

Variable	Value
Age, years	63 (56–70)
Male sex	70 (65%)
Creatinine, mg/dL	1.08 ± 0.3
Diabetes mellitus	24 (22%)
Hypertension	71 (66%)
Coronary artery disease	2 (2%)
Dyslipidemia	40 (37%)

Data are presented as mean ± standard deviation for normally distributed continuous variables, median (interquartile range) for non-normally distributed variables, and absolute values and percentages for categorical variables.

### Histological assessment of collagen volume fraction

The mean myocardial CVF in the study sample was 21.9 ± 5.5% (Table 2). Interstitial myocardial fibrosis was considered pathologically increased when CVF exceeded the mean plus 2 SD of values observed in a control group, corresponding to a reference value of 9.6%, as previously established.^16^ However, because mean CVF values in the present cohort were markedly higher than this reference, the median CVF was adopted as the cutoff for abnormal fibrosis (≥ 22.25%), resulting in 54 patients classified as having increased CVF. This threshold is consistent with clinically relevant values reported in prior research.^30,31^

**Table 2:** Fibrosis assessment parameters by preoperative cardiac magnetic resonance and histology

Parameter	Mean/median
LGE (%)	0 (0–1.74)
Global ECV without LGE (%)	29 ± 4.20
CVF (%)	22 ± 5.50

Data are presented as mean ± standard deviation for normally distributed continuous variables and median (interquartile range) for non-normally distributed variables. CVF: collagen volume fraction; ECV: extracellular volume; LGE: late gadolinium enhancement.

Using the reference value of 9.6%, 96% of patients (n = 104) in the total sample had increased CVF.

### Late gadolinium enhancement: macroscopic myocardial fibrosis

Regional myocardial fibrosis detected by LGE was present in 44% of patients (n = 47). The median percentage of LGE relative to total LV mass was 0 (IQR, 0–1.74), with a mean value of 1.3% in the overall sample (Table 2).

### Extracellular volume assessment: interstitial fibrosis

Mean ECV values measured in myocardial regions without LGE were 29 ± 4% (Table 2). Using a reference value of 30%, 32% of patients (n = 35) exhibited elevated ECV.

### Correlations between cardiac magnetic resonance-derived fibrosis measures and collagen volume fraction

Because of the asymmetric distribution of LGE values and the presence of outliers identified in exploratory analysis, Spearman correlation was used to assess the association between LGE and CVF. A moderate, statistically significant positive correlation was observed between LGE and CVF(p = 0.44; p < 0.001), indicating a monotonic association. A moderate correlation was also observed between ECV and CVF (r = 0.36; p = 0.001) (Table 3; Figure 7).

**Table 3:** Correlation between cardiac magnetic resonance-derived fibrosis parameters and collagen volume fraction

Parameter	ρ/r	p-value
Global LGE (%)	0.44	< 0.001
Global ECV without LGE (%)	0.36	0.001

CVF: collagen volume fraction; ECV: extracellular volume; LGE: late gadolinium enhancement.

**Figure 7:** Linear regression between LGE and CVF. CVF: collagen volume fraction; ECV: extracellular volume; LGE: late gadolinium enhancement.

### Diagnostic performance of cardiac magnetic resonance

The sensitivity of LGE for detecting histologically confirmed myocardial fibrosis was 64.8%, with a specificity of 77.8%, an accuracy of 71%, and an AUC of 0.73 (Table 4; Figure 8).

**Table 4:** Diagnostic performance of late gadolinium enhancement for detection of histological fibrosis

Parameter	Estimate (%)	95% CI
Sensitivity	64.80	50.60–77.30
Specificity	77.80	64.40–88.00
Accuracy	71.00	62.00–80.00
AUC	0.73	0.68–0.78

AUC: area under the receiver operating characteristic curve.

**Figure 8:** ROC curve of LGE for prediction of myocardial fibrosis by biopsy. CVF: collagen volume fraction; LGE: late gadolinium enhancement; ROC: receiver operating characteristic.

The diagnostic performance of ECV for identifying increased CVF (≥ 22.25%) showed a sensitivity of 40.7%, specificity of 74.1%, accuracy of 57%, and an AUC of 0.574. Agreement analysis using the Bland–Altman method demonstrated a mean bias of −0.167 (95% CI, −0.288 to −0.046). The limits of agreement displayed a trapezoidal pattern, indicating proportional bias and increasing variability with higher mean values. Cohen's kappa coefficient was low (0.171), indicating very weak agreement between ECV and histological fibrosis measures. These findings highlight important limitations in concordance that should be considered when interpreting ECV results (Table 5; Figure 9; Figure 10).

**Table 5:** Receiver operating characteristic (ROC) analysis of extracellular volume for prediction of histological myocardial fibrosis

Parameter	Estimate (%)	95% CI
Sensitivity	41	28–55
Specificity	74	60.30–85
Accuracy	57	48–67
AUC	0.57	0.49–0.66

AUC: area under the ROC curve.

**Figure 9:** Receiver operating characteristic (ROC) curve comparing CVF and ECV. AUC: area under the ROC curve; CVF: collagen volume fraction; ECV: extracellular volume.

**Figure 10:** Bland–Altman agreement analysis between CVF and ECV. CVF: collagen volume fraction; ECV: extracellular volume.

## Discussion

The findings of this study, summarized in Central Illustration, are consistent with prior research in demonstrating a substantial burden of myocardial fibrosis in patients with severe aortic valve disease. However, an important limitation relates to the biopsy sampling site. Tissue collection was restricted to the interventricular septum, resulting in a limited sample that may have constrained histopathological assessment, given that only a single myocardial fragment was obtained during surgery. Previous studies have reported stronger correlations between ECV and histology when multiple (n = 16) tissue blocks from different myocardial segments were analyzed, such as investigations using explanted hearts from patients with dilated or ischemic cardiomyopathy undergoing cardiac transplantation.^32^ In addition, myocardial fibrosis in aortic valve disease is not confined to the interventricular septum, which may further limit the representativeness of a single septal biopsy.^16^

The mean CVF observed in this cohort (22 ± 5.5%) reflects an advanced stage of interstitial remodeling. This finding is consistent with earlier studies indicating that chronic hemodynamic overload plays a central role in activating hypertrophic pathways and extracellular matrix deposition in aortic valve disease.^10,11^

Quantification of fibrosis by CMR showed moderate correlations between LGE and CVF (r = 0.44; p < 0.001), as well as between ECV and CVF (r = 0.36; p = 0.001). These results are directionally consistent with those reported by Azevedo et al.,^16^ who demonstrated a strong correlation between LGE and CVF (r = 0.69; p < 0.001). The lower magnitude of correlation observed in the present study may be explained by methodological differences in LGE quantification and by the higher proportion of patients with AS included in this cohort.

Macroscopic myocardial fibrosis detected by LGE was present in 44% of patients, supporting the concept that collagen deposition in advanced stages of aortic valve disease is heterogeneous and regionally distributed, as described by Nigri et al.^15^ However, LGE demonstrated a sensitivity of 64.8%, specificity of 77.8%, overall accuracy of 71%, and an AUC of 0.73 for the detection of histological fibrosis. These findings indicate that, although LGE is a valuable tool for identifying myocardial fibrosis, it may underestimate the total burden of interstitial fibrosis, particularly in patients without extensive focal collagen deposition.

ECV showed a moderate correlation with CVF, in contrast to a 2018 study that found no association between ECV and CVF, possibly because that cohort consisted exclusively of patients with AS, unlike the mixed valvular population in the present study. Nevertheless, ECV demonstrated lower diagnostic performance than LGE (AUC = 0.57), with a sensitivity of 40.7% and specificity of 74.1%. The reduced sensitivity of ECV may be partly attributable to fibrosis heterogeneity, as this technique quantifies expansion of the extracellular space without directly distinguishing collagen from other interstitial components. Bland–Altman analysis revealed a mean bias of −0.167 (95% CI, −0.288 to −0.046), indicating a slight underestimation of fibrosis by ECV. Agreement analysis yielded a low Cohen's kappa value (0.171), indicating only weak concordance between ECV and CVF.

Overall, T1 mapping–derived parameters highlight the role of CMR not only as a noninvasive diagnostic modality but also as a tool for advancing understanding of the pathophysiology of aortic valve disease, particularly mechanisms of myocardial remodeling and fibrosis formation. The moderate correlations observed between CMR-derived parameters and CVF support their applicability for myocardial fibrosis characterization and potentially for longitudinal assessment. Recent studies have also demonstrated associations between ECV and postoperative prognostic outcomes in patients with aortic valve disease, particularly when ECV is indexed to LV mass, which underscores the potential clinical relevance of these measures.^23^

## Conclusion

CVF is markedly increased in severe aortic valve disease, despite the sampling limitation inherent to biopsy collection restricted to the interventricular septum. These findings highlight the moderate specificity but low sensitivity of ECV for the detection of myocardial fibrosis, indicating that, although ECV may help exclude advanced fibrosis, its use as a standalone diagnostic marker for interstitial fibrosis remains limited.

Overall, our results reinforce that myocardial fibrosis is a multifactorial process and that imaging-based techniques have inherent limitations in its detection and quantification. A combined assessment using LGE and ECV may provide complementary information, particularly for the early identification of interstitial fibrosis. Further studies are warranted to determine the prognostic impact of these measures on the clinical course of aortic valve disease and to evaluate the potential of T1 mapping as a more sensitive marker for early myocardial fibrosis. This study underscores the relevance of CMR in the assessment of myocardial fibrosis and microstructural remodeling, with potential implications for therapeutic decision-making and optimal timing of intervention.

## Data Availability

The underlying content of the research text is contained within the manuscript.
